# Secondary B Cell Receptor Diversification Is Necessary for T Cell Mediated Neuro-Inflammation during Experimental Autoimmune Encephalomyelitis

**DOI:** 10.1371/journal.pone.0061478

**Published:** 2013-04-22

**Authors:** Georgina Galicia, Bryant Boulianne, Natalia Pikor, Alberto Martin, Jennifer L. Gommerman

**Affiliations:** Department of Immunology, University of Toronto, Toronto, Ontario, Canada; Escola Paulista de Medicina – UNIFESP, Brazil

## Abstract

**Background:**

Clinical studies of B cell depletion in Multiple Sclerosis (MS) have revealed that B Lymphocytes are involved in the neuro-inflammatory process, yet it remains unclear how B cells can exert pro- and anti-inflammatory functions during MS. Experimental Autoimmune Encephalomyelitis (EAE) is an animal model of MS whereby myelin-specific T cells become activated and subsequently migrate to the Central Nervous System (CNS) where they perform pro-inflammatory functions such as cytokine secretion. Typically EAE is induced by immunization of mice of a susceptible genetic background with peptide antigen emulsified in Complete Freund's Adjuvant. However, novel roles for B-lymphocytes in EAE may also be explored by immunization with full-length myelin oligodendrocyte glycoprotein (MOG) that contains the B cell conformational epitope. Here we show that full length MOG immunization promotes a chronic disease in mice that depends on antigen-driven secondary diversification of the B cell receptor.

**Methods:**

Activation-Induced Deaminase (AID) is an enzyme that is essential for antigen-driven secondary diversification of the B cell receptor. We immunized AID^−/−^ mice with the extracellular domain (amino acids 1–120) of recombinant human MOG protein (rhMOG) and examined the incidence and severity of disease in AID^−/−^ versus wild type mice. Corresponding with these clinical measurements, we also evaluated parameters of T cell activation in the periphery and the CNS as well as the generation of anti-MOG antibodies (Ab).

**Conclusions:**

AID^−/−^ mice exhibit reduced severity and incidence of EAE. This suggests that the secondary diversification of the B cell receptor is required for B cells to exert their full encephalogenic potential during rhMOG-induced EAE, and possibly also during MS.

## Introduction

It has been long recognized that B cells are present in the MS central nervous system (CNS) [1], [2], including white matter lesions [3], [4], the normal appearing white matter [5], the cerebrospinal fluid (CSF) [6], [7], perivascular spaces in the CNS [3], and the CNS meninges [8]. In addition, clinical trial results with B cell depleting agents Rituximab and Ocrelizumab [9], [10] support a critical role for B-lymphocytes in MS pathology. However the mechanism of how B cells contribute to MS disease, and which B cell subsets are pathogenic versus anti-inflammatory remains unclear.

B cells have varying roles in Experimental Autoimmune Encephalomyelitis (EAE), an animal model of MS, depending on which EAE model is employed. The MOG_35-55_ C57Bl/6 EAE model has been used to demonstrate that B cell deficient mice exhibit similar clinical incidence of EAE as WT counterparts [11], although B cell depletion before and during MOG_35-55_ C57Bl/6 EAE can have profound effects on clinical disease, in particular due to the loss of regulator IL-10-secreting B cells [12], [13]. An alternative model to MOG_35-55_ C57Bl/6 EAE is the induction of EAE with full-length myelin proteins that contain B cell conformational epitopes [14]. For example, mice immunized with human recombinant MOG_1-120_ (rhMOG), the conformational extracellular portion of MOG that is accessible on the surface of the myelin sheath, develop EAE that is dependent on B cells [15], [16]. Similarly, B cells are required for neuroinflammation when mice are immunized with a chimeric fusion protein of two auto-Ags (myelin basic protein - MBP and proteolipid protein - PLP) [17].

In terms of which types of B cells enter the CNS during MS, the majority exhibit a memory phenotype (CD19^+^CD27^+^CD138^−^), and in this location, an oligoclonal B cell repertoire has been observed that may be linked to the specificities of the intrathecal IgGs that are observed in the CSF during MS [18]–[22]. The expression of CD27 on these intrathecal B cells suggests a post-germinal centre (GC) phenotype, and indeed evidence suggests that some CNS-resident B cells have undergone class switch recombination (CSR) and somatic hypermutation (SHM) of their B cell receptor (BCR) [23]–[25]. As such, secondary diversification of the BCR may be important for the etiopathology of MS.

B cells deficient in AID cannot undergo CSR nor SHM as these secondary BCR diversification processes are absolutely dependent on the enzymatic activity of AID [26], [27]. AID is a DNA-specific cytosine deaminase that triggers SHM and CSR by deaminating deoxycytosine to deoxyuridine within Ig genes [28]–[31]. DNA repair pathways then act on the uridines created by AID resulting in the generation of point mutations in the V-region or recombinogenic events that lead to CSR [30]. B cells with these point mutations are then assessed for their ability to bind Ag with high affinity in the competitive environment of the GC within secondary lymphoid tissues (lymph nodes, spleen). AID^−/−^ mice immunized with MOG_35-55_ peptide in adjuvant have been shown to exhibit normal clinical symptoms of EAE [32]. However, the role of BCR secondary diversification has not been tested in the context of immunization with MOG Ag that contains the conformational epitope. In this report, we re-examined the role of BCR secondary diversification in EAE by testing the effect of immunizing AID^−/−^ mice with rhMOG.

## Results

### AID-deficient mice exhibit impaired EAE in response to rhMOG immunization

**Figure 1:**
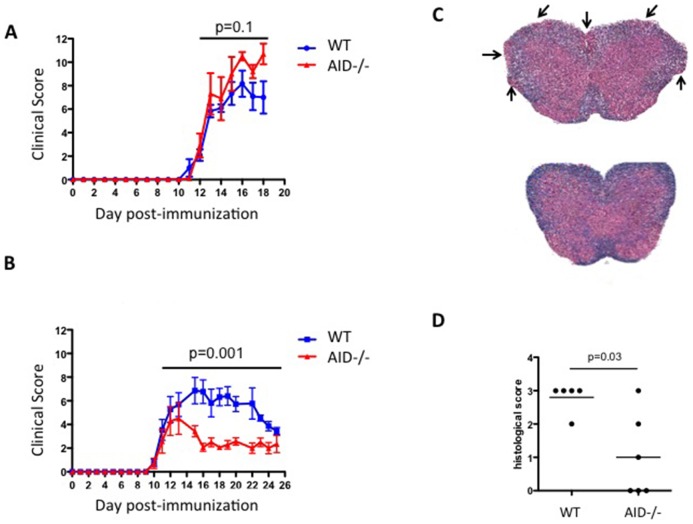
AID^−/−^ mice exhibit attenuated EAE in response to rhMOG but not MOG_35-55_. (**A**) WT and AID^−/−^ mice were immunized with MOG_35-55_ in CFA and examined for clinical symptoms. A representative experiment is shown, and 2 experiments were performed with similar results (shown here are n = 6 WT and n = 5 AID^−/−^ mice per group). (**B**) WT and AID^−/−^ mice were immunized with rhMOG in CFA and examined for clinical symptoms. A representative experiment is shown (see Table 1 for all 4 experiments) with n = 11 WT and n = 7 AID^−/−^ mice per group. At the termination of the experiment, spinal cords were dissected and stained with H&E and counterstained with Luxol fast blue. A representative example of 6 separate mice is shown (original magnification ×200). See arrows for areas of cellular infiltrates (**C**). Cell infiltration in the spinal cord tissue was assessed as follows: meningeal infiltrate (score 1), perivascular infiltrate (score 2), parenchymal infiltrate (score 3) (**D**).

To examine the role of BCR secondary diversification during EAE, we immunized AID^−/−^ and WT mice with either MOG_35-55_ peptide, or full-length MOG_1-120_ (rhMOG), emulsified in Complete Freund's Adjuvant followed by 2 injections of pertussis toxin (see Materials and Methods for details). Consistent with earlier reports [32], the incidence and severity of disease provoked by MOG_35-55_ peptide was similar between WT and AID^−/−^ mice (Fig. 1A, no statistically significant difference during the disease period up to day 18 by ANOVA). In contrast, compared to WT mice, the incidence and severity of disease provoked by rhMOG was significantly reduced in AID^−/−^ mice throughout the chronic stage of the disease (Fig. 1B, Table 1). The milder EAE clinical symptoms in AID^−/−^ mice was accompanied by less infiltration of leukocytes into the spinal cord, and a diminishment in CNS pathology compared to WT mice (Fig. 1C–D). Thus, while MOG_35-55_ peptide induced EAE in AID^−/−^ mice (Fig. 1A and [32]), immunization with rhMOG results in less severe clinical disease [32].

**Table 1:** Summary of EAE experiments.

Experiment	Genotype	Incidence (%)	Day of onset	peak score	Average cumulative score	ANOVA
**1**	WT (n = 8)	100	10	12.06±0.62	55.8±6.5	p = 0.001
	AID^−/−^ (n = 12)	81	11	4.58±2.77	19.7±10.8	
**2**	WT (n = 15)	100	11	7.167±2.54	28.8±9.7	p = 0.001
	AID^−/−^ (n = 15)	66	11	2.7±0.6	9.7±6.8	
**3**	WT (n = 6)	100	11	7.7±3.9	25.5±16.8	p = 0.001
	AID^−/−^ (n = 6)	33	11	0.58±1.42	8.3±16.5	
**4**	WT (n = 8)	100	10	10.19±0.1	32.7±3.1	P = 0.0001
	AID^−/−^ (n = 9)	33	13	1.7±2.1	4.7±3.4	

### T cell priming to rhMOG in the draining lymph node is unaffected in AID^−/−^ mice

**Figure 2:**
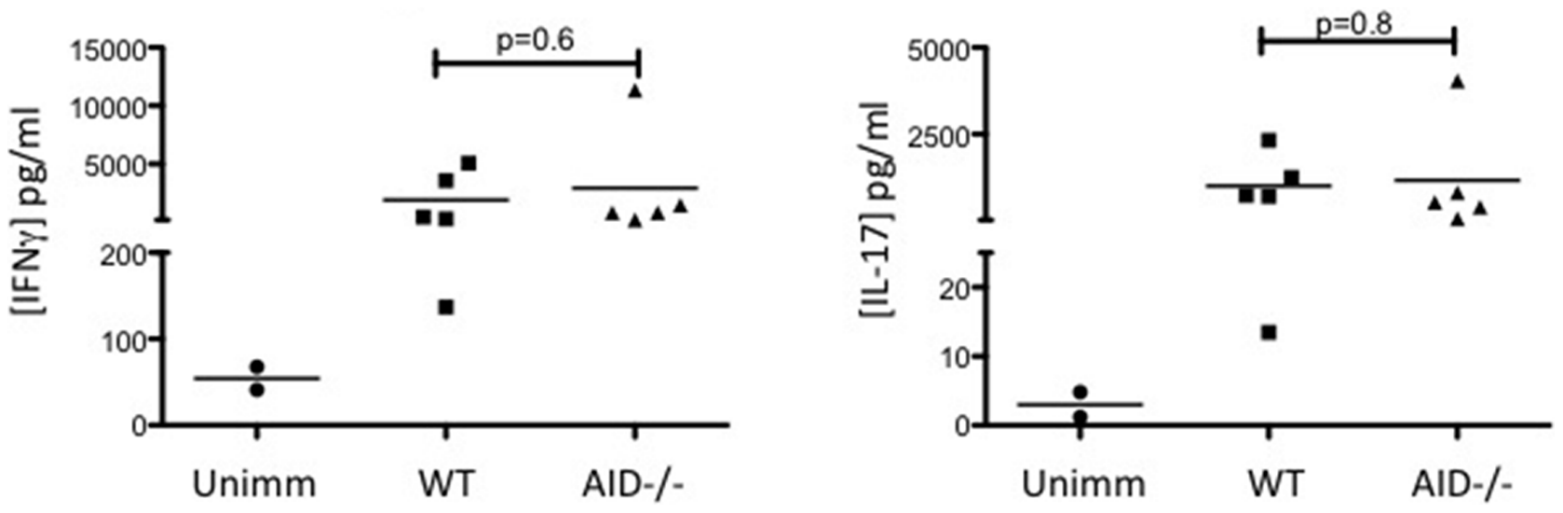
Lymph node cells from WT and AID^−/−^ mice produce equivalent levels of cytokines in response to immunization with rhMOG. WT and AID^−/−^ mice were immunized with recombinant human MOG (rhMOG) and draining axillary and brachial lymph nodes were harvested after 7 days post-immunization. Four million lymph node cells were plated along with 20 µg of rhMOG. Cultures were kept for 48 hours after which the supernatant was harvested and evaluated for cytokine production by ELISA. The limit of detection of the assay is 125 pg/ml for the IFNγ ELISA and 63 pg/ml for the IL-17 ELISA. Five mice per group were assessed.

Since B and T lymphocytes interact intimately within the secondary lymphoid tissue during an immune response, and B cells can function as antigen-presenting cells, the lack of secondary diversification of the BCR of AID^−/−^ B cells may have altered T cell priming to rhMOG immunization. To test this, we isolated leukocytes from the draining lymph nodes 7 days post-immunization with rhMOG. This time point precedes disease onset and provides an opportunity to separate the priming phase of the disease from the effector phase whereby T cells migrate to the CNS and secrete pro-inflammatory cytokines. Draining lymph node cells from WT and AID^−/−^ mice were cultured with rhMOG *in vitro* for 2 days in order to allow for Ag processing and presentation, at which point supernatants were harvested from these cultures and tested for the presence of both IFNγ and IL17 by ELISA. Both cytokines were readily detected in the supernatants of immunized WT and AID^−/−^ mice, but not in the supernatants of lymph node cell suspensions from unimmunized mice, and no significant differences in the amounts of IFNγ and IL17 in the supernatants of WT and AID^−/−^ cell cultures were observed (Fig. 2). To ascertain if these cytokines were derived from CD4^+^ T cells, we performed intracellular FACS analysis. Both WT and AID^−/−^ CD4^+^ T cells produced low levels of IFNγ and IL17 in response to a 48-hour re-stimulation *ex vivo* with rhMOG (Supplemental Fig. S1). Therefore, the priming of CD4^+^ T cells to MOG Ag appears to be normal in AID^−/−^ mice, and differences during the rhMOG priming phase likely do not account for the reduced clinical severity of EAE.

### Accumulation of cytokine producing CD4^+^ T cells in the CNS is impaired in AID^−/−^ mice

**Figure 3:**
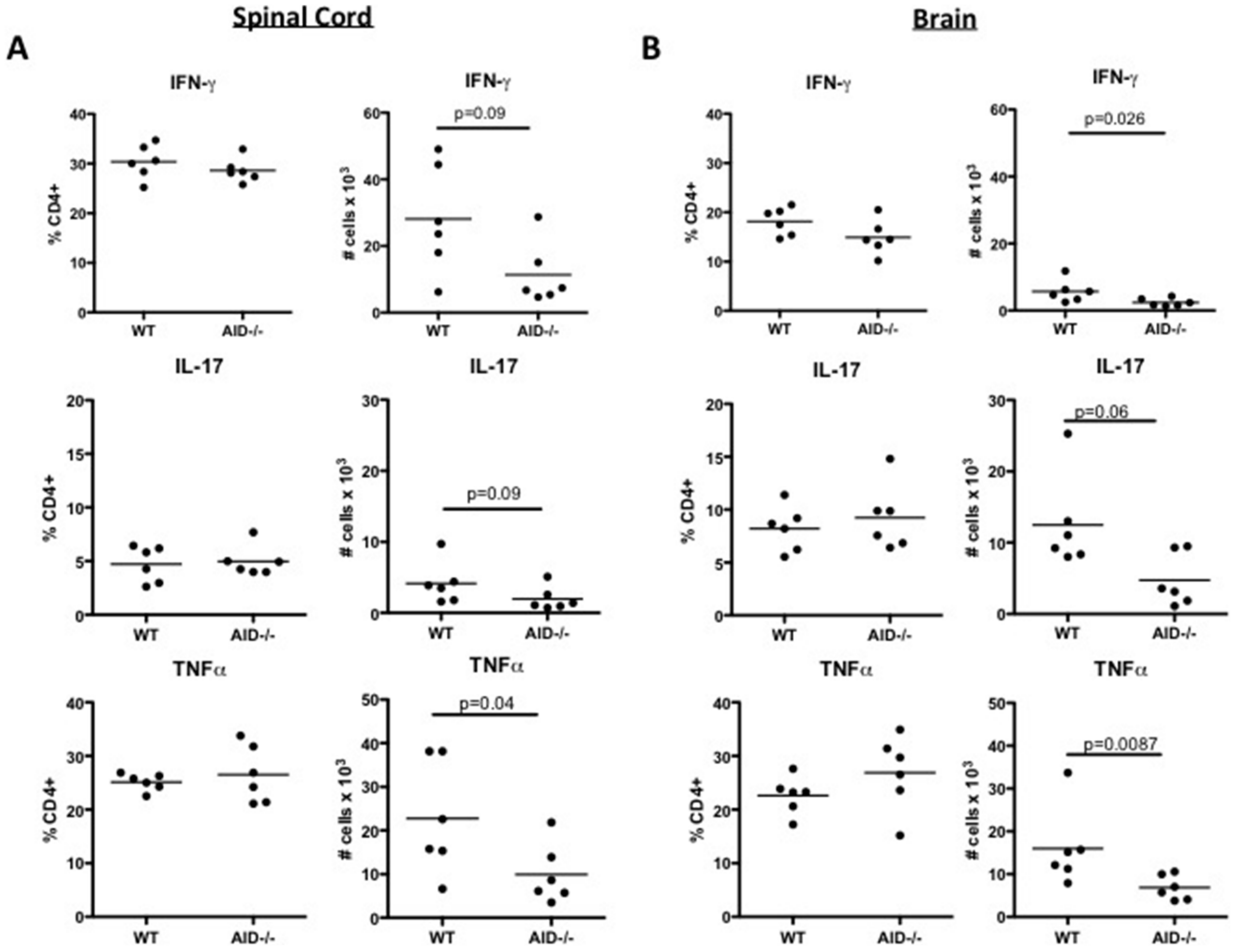
Impaired accumulation of cytokine producing CD4^+^ T cells in the CNS of AID^−/−^ mice. WT and AID^−/−^ mice were immunized with recombinant human MOG (rhMOG) and spinal cords (**A**) and brains (**B**) were extracted and processed at day 15: the peak of disease. Leukocytes were stimulated *ex vivo* with PMA/Ionomycin, and brefeldin A was added in the last 4 hours. Cells were then subjected to surface and intracellular cytokine staining. This experiment was performed twice with similar results and 6 mice per group were tested in this experiment. Representative FACS analysis can be seen in Supplemental Figure S2A.

Following priming in the draining lymph node, activated MOG-primed CD4^+^ T cells migrate to the CNS where they are re-stimulated by local antigen-presenting cells to produce cytokines such as IFNγ, IL17 and TNFα. To examine if these events were dependent on B cells that have undergone secondary diversification of the BCR, single cell suspensions of spinal cord and brain derived CD4^+^ T cells were subjected to surface staining and intracellular cytokine FACS analysis (see Supplemental Fig. S2A for representative FACS). The frequency of IFNγ^+^, IL17^+^ and TNFα^+^ CD4^+^ T cells as a percentage of the total CD4^+^ T cell pool was comparable between WT and AID^−/−^ mice, although a reduced number of IFNγ^+^, IL17^+^ and TNFα^+^ CD4^+^ T cells was observed in the spinal cord (Fig. 3A, trending for IFNγ and IL17, statistically significant for TNFα) and the brain (Fig. 3B trending for IL17, statistically significant for IFNγ and TNFα) of AID^−/−^ mice compared to WT controls. Therefore, optimal accumulation of cytokine-producing CD4^+^ T cells in the CNS of mice with EAE requires the expression of AID in B cells.

### AID^−/−^ B cells produce anti-MOG IgM and accumulate in the CNS of EAE mice

**Figure 4:**
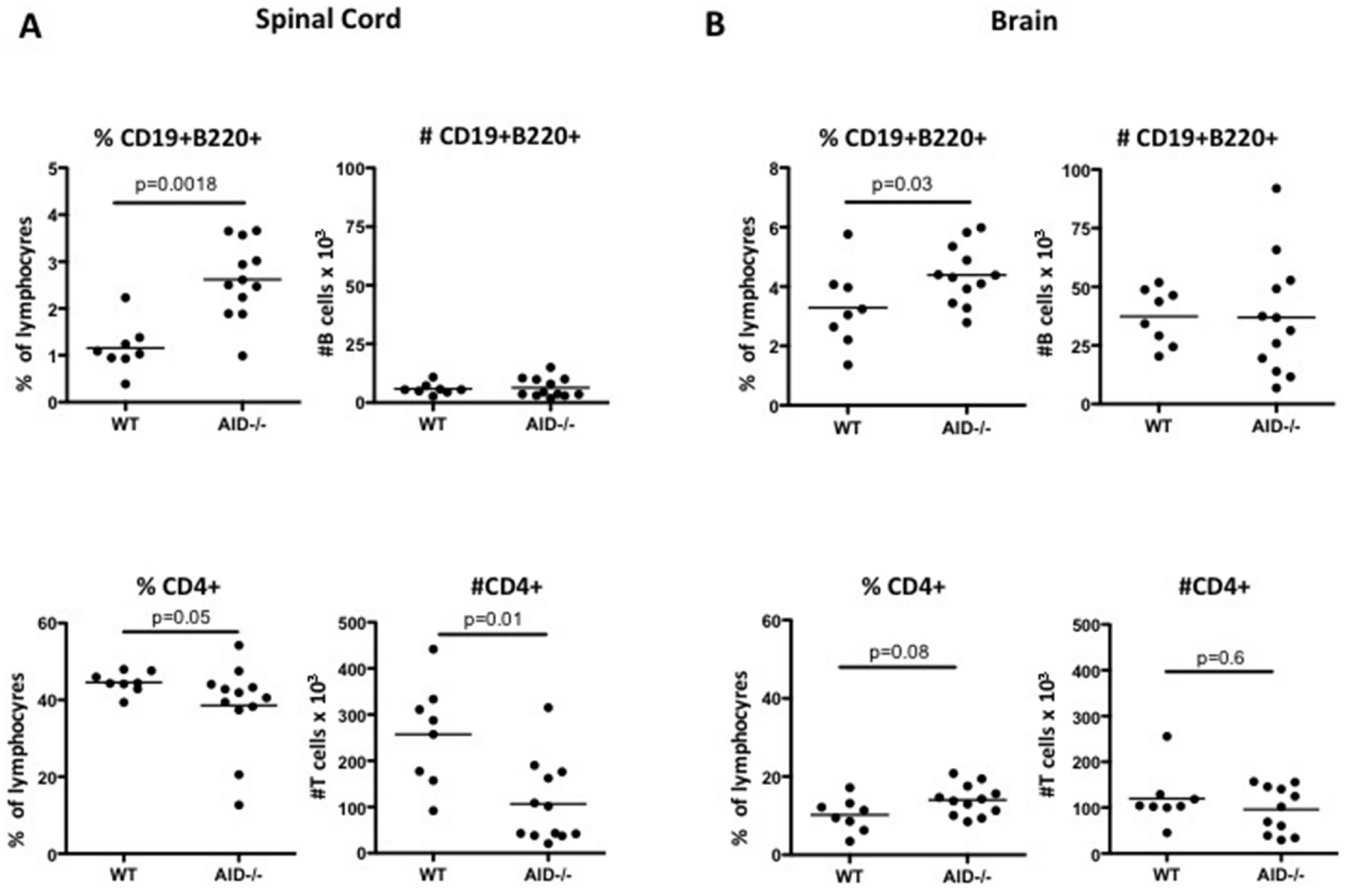
AID^−/−^ B cells accumulate normally in the CNS. WT and AID^−/−^ mice were immunized with recombinant human MOG (rhMOG) and spinal cords (**A**) and brains (**B**) were extracted and processed at day 15 (peak of disease). B cells and T cells were quantified by flow cytometry. Although the frequency of B cells was observed to be greater in AID^−/−^ mice, the absolute numbers of B cells was unaffected. In contrast, T cell frequency and numbers were reduced in the spinal cord. This experiment was performed twice with similar results and 8–12 mice per group were tested.

**Figure 5:**
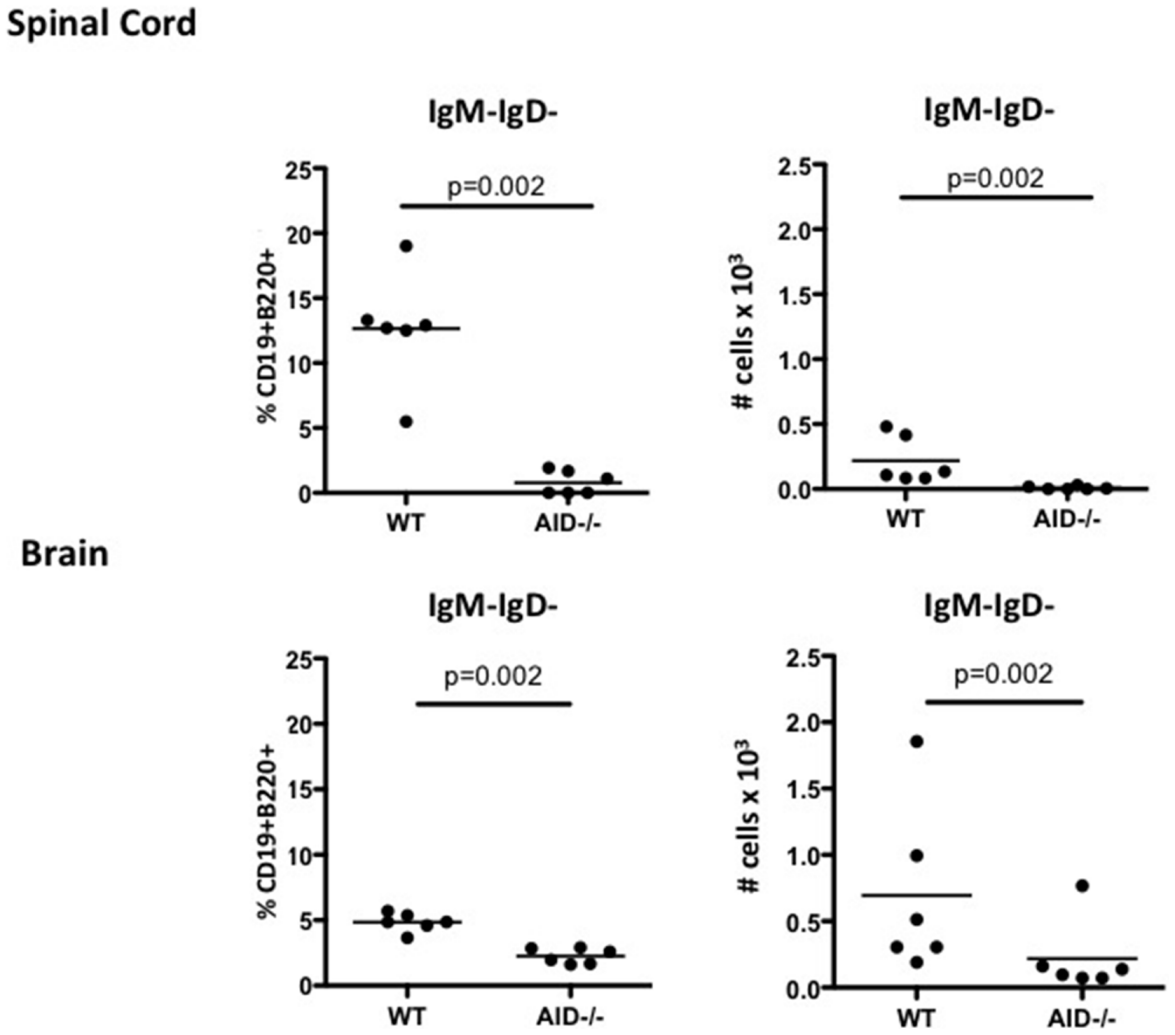
Appearance of class switched B cells in the CNS during EAE. WT and AID^−/−^ mice were immunized with recombinant human MOG (rhMOG) and spinal cords were extracted and processed at day 15 (peak of disease). B cells were identified by the expression of CD19 and B220. Class switched B cells were further identified by the absence of IgM/IgD and reported here as a percentage of the total number of B220^+^CD19^+^ B cells. Representative FACS analysis can be seen in Supplemental Figure S2B.

CD19^+^B220^+^ B cells were also detected in the CNS of rhMOG immunized mice at the peak of disease (day 15), and when reported as a frequency, a statistically significant elevation in B cells was observed in the brain and spinal cord of AID^−/−^ mice (Fig. 4A,B). However, when absolute numbers of B cells were enumerated, B cell numbers in WT brain and spinal cord were roughly equivalent to those in the brain and spinal cord of AID^−/−^ mice (Fig. 4A,B). Therefore, although B cells migrate in equal numbers to the CNS of AID^−/−^ and WT mice, there is less infiltration of other leukocyte cell types into the brain and spinal cord of AID^−/−^ mice thus accounting for the over-representation of B cells within the AID^−/−^ CNS. Indeed the number and frequency of CD4^+^ T cells was reduced in the AID^−/−^ spinal cord (Fig. 4A,B). This reduction in T cells may partially account for the over-representation of B cells in the spinal cord, although other inflammatory cells in addition to T cells may also be decreased. Examination of the phenotype of CNS-resident CD19^+^B220^+^ B cells revealed that a proportion of B cells in the WT CNS had class switched their BCR at the peak of disease, and not surprisingly, class-switched AID^−/−^ B cells are not observed in the CNS during EAE (Fig. 5A - enumeration of IgM^−^IgD^−^ B cells is shown, see also representative FACS plots in Supplemental Fig. S2B). Therefore, CSR or SHM of the BCR is not a pre-requisite for migration to the CNS during EAE.

**Figure 6:**
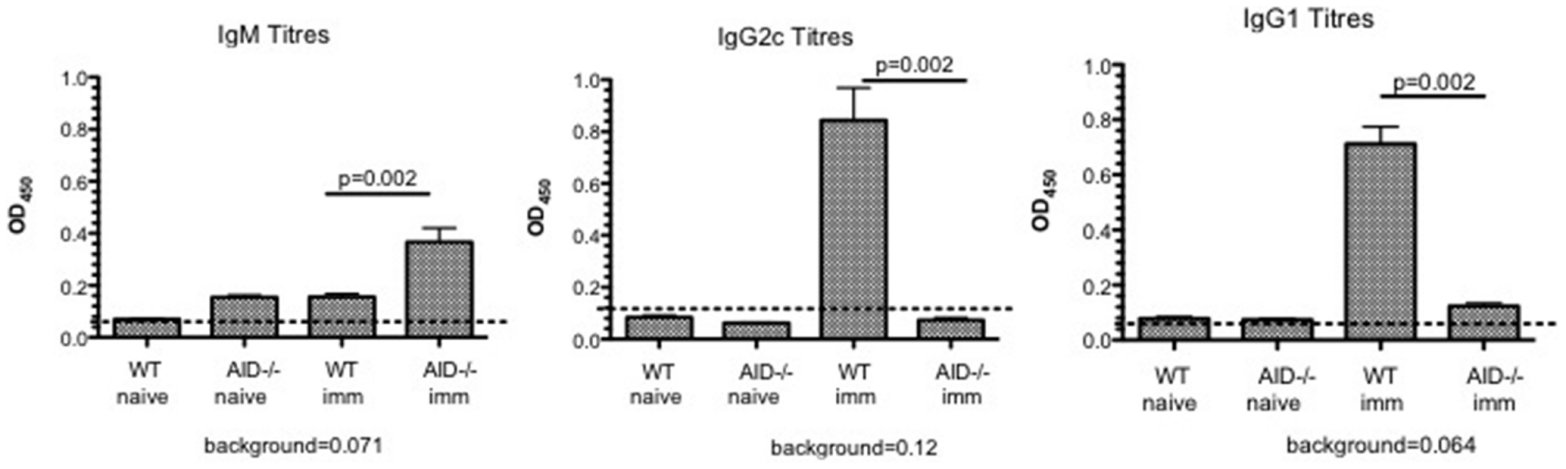
Anti-MOG antibodies generated in WT and AID^−/−^ mice. WT and AID^−/−^ mice were immunized with recombinant human MOG (rhMOG) and titres of anti-MOG Ab were evaluated at day 15 (peak of disease). As expected, AID^−/−^ mice only made anti-MOG Ab of the IgM isotype, whereas WT mice produced anti-MOG IgG1 and IgG2c Ab (and very little IgM at this time point). The average OD for each mouse at a set concentration of serum (half-way point of the curve) is depicted, and raw OD values can be seen in Supplemental Figure S3. In this experiment, 5–6 mice per group were tested and an additional experiment tested 8–12 mice.

Consistent with the appearance of class switched B cells in the WT CNS, significant serum titres of anti-MOG IgG1 and IgG2c Ab were detected at the peak of disease, although at this time point very little anti-MOG IgM was detected (Fig. 6). In contrast, AID^−/−^ mice exhibited anti-MOG IgM titres, and not surprisingly, an absence of detectable class switched (IgG1, IgG2c) anti-MOG Ab (Fig. 6). Collective O.D. measurements for these titres across a range of concentrations can be seen in Supplemental Fig. S3. Collectively, these data show that rhMOG immunization provokes the production of class switched anti-MOG Ab and the appearance of class switched B cells in the CNS, and these processes are impaired in AID^−/−^ mice.

## Discussion

In this study we now show that in the absence of AID, EAE incidence and severity in response to rhMOG immunization is significantly reduced. B cells have been previously shown to be important in MS and in rhMOG-induced EAE [15], [16]. Since AID expression is restricted to B cells [33] and is necessary for SHM and CSR [26], [27], we conclude that the presence of un-switched, un-mutated B cells is not sufficient to propagate neuroinflammation in response to rhMOG immunization. We have also shown that expression of AID in B cells is not required for the optimal priming of MOG-specific T cells in the periphery by measuring cytokines by ELISA (Fig. 2). Intracellular staining of IFNγ, IL17 and TNFα by FACS confirmed these findings, although this assay was less sensitive than the ELISA readout, presumably because we did not necessarily capture the moment in culture where cytokine secretion by CD4^+^ T cells was optimal (Supplemental Fig. S1). Nevertheless, our finding that WT and AID^−/−^ mice exhibit equivalent CD4^+^ T cell priming to MOG Ag is consistent with the observation that priming of MOG-specific T cells is also normal in B cell deficient mice [15], suggesting that other leukocytes such as Dendritic Cells are sufficient for T cell priming in the periphery.

In contrast to our findings that T cell priming was normal in the peripheral lymph nodes prior to disease onset, we found that the accumulation of cytokine secreting CD4^+^ T cells in the CNS was compromised in AID^−/−^ mice. The reduction in cytokine secreting CD4^+^ T cells in the AID^−/−^ CNS is likely due to the observed diminished recruitment of CD4^+^ T cells to the CNS. However, in one series of experiments we noted that in the context of very strong inflammation (peak clinical scores greater than 10 for WT mice), the frequency of cytokine-producing T cells was also significantly reduced in the spinal cord, in particular the frequency of CD4^+^ T cells that produce multiple cytokines (Supplemental Fig. S4). Such differences were not observed consistently in the brain, although in general there were fewer cytokine-producing cells in this location (data not shown). Thus, in situations of very robust EAE, an additional defect in the polarization of CD4^+^ T cells in the spinal cord was observed in AID^−/−^ mice, suggesting that Ag presentation within the spinal cord may be compromised in the absence of B cells with a switched and/or mutated BCR.

In response to rhMOG immunization, significant titres of anti-MOG IgG1 and IgG2c Ab are observed in WT but not AID^−/−^ mice. Although we report a very low percentage of class-switched B cells in the CNS of WT mice (approximately 7–15% of total B cells in the spinal cord are IgM^−^IgD^−^), we detect very low titres of anti-MOG IgM Ab in these mice. This suggests that the majority of IgM-expressing B cells in the CNS are not secreting anti-MOG Ab. It is possible that these are not MOG-specific B cells but rather bystander B cells that migrate to the CNS in response to inflammation. Alternatively, these may represent B cells that are producing Ab to *de novo* CNS Ag, a phenomenon we have observed in a sub-strain of SJL/J mice with EAE [34].

In the rhMOG model, transfer of Ab from rhMOG, but not MOG_35-55_ immunized mice is capable of rescuing disease in B cell deficient mice [35], suggesting that rhMOG-driven Ab exerts pathogenic functions during EAE. The exact mechanism by which anti-MOG Ab instigate EAE remains unclear, and further experiments testing the pathogenic potential of class-switched anti-MOG Ab in AID^−/−^ recipient mice would shed further light on this question. Indeed, since IgG Ab raised against rhMOG specifically can bind to oligodendrocytes, it presumably exerts pathogenic activity during EAE [36]. In addition, recent results from Sun et al, which corroborate our findings that AID^−/−^ mice exhibit reduced EAE severity, suggest that serum IgM Ab derived from EAE mice binds to CNS tissue in a different pattern than serum IgG derived from WT EAE mice [37]. Therefore the quality of Ab generated by B cells during EAE may be directly or indirectly required for exacerbating CNS pathology in mice.

On the other hand, although in the case of the human disease humoral involvement in MS has been supported by the presence of oligoclonal bands (OCBs) of Ab in MS patient cerebral spinal fluid [38], OCBs are not unique to MS and are also detected in other inflammatory neurological diseases [39]–[41]. Furthermore, the finding that OCB were not decreased in B cell depleted MS patients that exhibited a positive clinical response to the therapy suggests that Ab in the CSF may not be related to disease activity [9]. Therefore, the AID^−/−^ rhMOG model may be useful for dissecting the relative role of anti-MOG Ab in mice where B cells are still present. Indeed, given that AID has been implicated in systemic autoimmunity in an Ab-independent manner [42], further study of rhMOG immunized AID^−/−^ mice will be important for determining Ab-dependent and Ab-independent roles for B cells during EAE.

Switched B cells are observed in low frequencies in the CNS during EAE (Fig. 5), thus it is plausible that a class-switched and/or hyper-mutated BCR may enhance polarization of local T cells through B cell - T cell cross-talk. Alternatively, the observed reduction in cytokines produced by T cells in the AID^−/−^ spinal cord may be a consequence of local suppression of MOG-specific T cells by CD40/IL21 driven “B-regulatory” cells that have been shown to dampen neuroinflammation [43]. Indeed, CD40 signals are abundant in the GC compartment, and we have previously shown that AID^−/−^ B cells accumulate within the GC [44]. Therefore, it is possible that in the context of chronic inflammation, excessive CD40 signaling (in the absence of CSR/SHM) could divert B cells towards a more regulatory phenotype. Further experiments whereby CNS-derived AID^−/−^ B cells are transferred into WT mice would potentially provide further mechanistic insights into these putative scenarios.

In summary, using the rhMOG EAE model, we have uncovered a novel role for B cells that have undergone secondary diversification of their BCR in the neuroinflammatory cascade.

Since total B cell ablation has risks for the patient, selective targeting of Ag-experienced (class switched, hypermutated) B cells may represent an alternative strategy for the treatment of MS patients.

## Materials and Methods

### Ethics Statement

Experiments that used animals for this manuscript were approved by the ethical committee for animal experimentation of the Faculty of Medicine of the University of Toronto (Canada) following international guidelines (Accreditation number of the laboratory: 20009017)

### Mice and EAE induction

Wild-type (WT) and AID^−/−^ mice bred on the C57BL/6 background (obtained from Charles River Laboratories and Tasuku Honjo, Kyoto University, Kyoto, Japan, respectively) used for EAE induction with rhMOG. EAE was induced by immunization with 100 µg of rhMOG or 100 µg of MOG_35-55_ peptide emulsified 1∶1 in Complete Freund's Adjuvant (Sigma) containing 4 mg/ml of *Mycobateria tuberculosis* (H37RA) (Difco). The amount of rhMOG was pre-determined based on earlier reports [35], [45]. Mice also received two intra-peritoneal doses of pertussis toxin (200 ng) (List biological laboratories Inc.) the day of immunization and 48 h later. Mice were weighed daily and the clinical disease was assessed with a modified scale derived from Giuliani and colleagues [46]. To the Giuliani scale we added the assessment of the righting reflex. The righting reflex capacity was graded from 0 to 2. Zero was assigned for a normal righting reflex, 1 for slow righting reflex, and 2 for a delay of more than 5 seconds in the righting reflex. Thus, the modified scale ranges from 0 (no-symptoms) to 16 (fully quadriplegic mouse with limp tail and significantly delayed righting reflex). All experiments were performed according to our University of Toronto approved animal-use protocols. Specifically, mice that show evidence of partial hind limb paralysis were given supplemental mash and sub-cutaneous saline injections of 1 ml–1.5 ml of lactated Ringer's solution, 1–2 times daily depending on symptoms. In addition, mice were monitored twice daily instead of once per day. Mice that received a score of 15 were not permitted to remain as a score of 15 for greater than 24 hours. Mice that appeared moribund or had exhibited a weight loss of greater than 20% were euthanized promptly and assigned a score of 16. Euthanasia was by CO_2_ over-dose. Death was not an endpoint. Signs of general malaise (hunched back, weight loss >20%, ruffled coat, sunken eyes) were also monitored, and irrespective of EAE score, these animals were given supplemental mash, sub-cutaneous saline injections of 1 ml–1.5 m of lactated Ringer's solution, 1–2 times daily depending on symptoms, and monitored twice daily. In general, animals did not exhibit these symptoms in the absence of limb paralysis.

### Preparation of rhMOG

The rhMOG protein used to immunize our mice consists of 120 amino acids of the extracellular portion of human MOG. An *E. coli* strain that produces rhMOG was obtained from Drs. Chris Linington and Nancy Ruddle. rhMOG was expressed by bacteria in a soluble form and subsequently purified from the bacterial supernatant using a Ni^2+-^His-bind resin column (Novagen). The purified rhMOG protein was analyzed by SDS-PAGE using a 15% polyacrylamide gel stained with Coomassie blue and confirmed to be pure and of the appropriate molecular weight.

### Assessment of immunopathology in the spinal cord

Immune-mediated inflammation was analyzed at day 14 post-immunization. Briefly, at the time of sacrifice mice were perfused with ice-cold PBS. Spinal cords intended for histology were subsequently fixed in formalin and embedded in paraffin. Paraffin-embedded tissues were subsequently stained with Luxol fast blue and H&E.

### Flow cytometry

Fourteen days post-immunization mice were sacrificed and intra-cardially perfused with 30 ml of ice-cold PBS. Brain and spinal cord were harvested and disaggregated in HSSB containing 1M HEPES, 5 M NaCl, 1M MgCl_2_, 1M KCl, and 1M of CaCl_2_ in the presence of 1 mg/ml of collagenase D (Roche Diagnostics) and 60 µg/ml of DNase (Roche Diagnostics). Samples were filtered through a 70 µm cell strainer (BD Falcon) to obtain a single cell suspension. Digested tissue was re-suspended in 30% Percoll solution and centrifuged 20 min at 2000 rpm. A pellet containing mononuclear cells was re-suspended in FACS buffer (10% FBS, 0.02% NaN_3_ PBS). Cells were labeled with anti-CD4, anti-B220, anti-CD19, anti-IgM, and anti-IgD (all eBioscience, San Diego, CA). For intracellular cytokine detection, cells were stimulated with phorbol 12-myristate 13-acetate (50 ng/ml; Sigma) and ionomycin (0.5 mg/ml; Sigma) and Brefeldin A for 4 h. After stimulation, cells were stained extracellularly with anti-CD4, the cells were fixed and permeabilized with Perm/Wash solution (BD Biosciences Mississauga, ON Canada), and finally cytokines were detected with anti-IL17, anti-TNFα, and anti-IFNγ Ab (eBiosciences Mississauga, ON Canada). Staining was analyzed using FACScanto flow cytometer and FlowJo software.

### Assessment of rhMOG priming in the draining lymph nodes

Mice were immunized as for EAE induction. Seven days after immunization draining lymph nodes and spleen cells were harvested and single-cell suspensions were prepared by passage through a cell strainer (BD Biosciences). Draining lymph node cells cells (3×10^5^) and splenocytes (5×10^5^) were cultured in the absence or presence of rhMOG (20 µg/mL) in 24-well plates. Supernatants were recovered after 48 h of culture. IFNγ (ELISA kit from Invitrogen) and IL17A (ELISA ready set-Go ebioscience) were quantified by sandwich ELISA as per the manufacturer's directions.

#### Measuring anti-MOG Ab titres

rhMOG-specific Ab were analysed by ELISA methodology by coating 96-well plates (Nunc) overnight at 4 C with 1 µg/ml of MOG_35-55_ peptide in PBS. Wells were blocked with PBS 2% BSA for 2 h at room temperature. Plates were washed 4 times with PBS 0.05% Tween-20. Serial dilutions of each serum sample were added at 100 µl/well in duplicate and incubated for 2 h at room temperature. 1 µg/ml of anti-IgG1-biotin, anti-IgG2c-biotin, or IgM-biotin (100 µl/well) (BD) were subsequently added and incubated 1.5 h at room temperature. After washing the plates four times with PBS 0.05% Tween-20, horseradish peroxidase conjugated streptavidin was added (R&D systems). Plates were incubated 30 min and bound MOG-specific Ab were detected with 3,30,5,50-Tetramethylbenzidine (TMB). Optical density was read at 450 nm.

### Statistical analysis

Statistical analyses were performed with GraphPad Prism (GraphPad Software Inc., San Diego, USA) by using the non-parametric two-tailed Mann-Whitney U test or in the case of EAE disease course, we used ANOVA. A difference was considered to be significant when p<0.05.

## Supporting Information

Figure S1 **Lymph node CD4^+^ cells from WT and AID^−/−^ mice both produce cytokines in response to immunization with rhMOG.** WT and AID^−/−^ mice were immunized with recombinant human MOG (rhMOG) and draining axillary and brachial lymph nodes were harvested after 7 days post-immunization. 4 million lymph node cells were plated along with 20 µg of rhMOG. Cultures were kept for 48 hours, then were harvested and subjected to intracellular cytokine staining. Shown here are cells pre-gated based on live staining and surface expression of CD4. A representative example is shown and 5 mice per group were tested, with the experiment performed twice with similar results.
(TIF)

Figure S2 **Representative FACS of CNS leukocytes.** WT and AID^−/−^ mice were immunized with recombinant human MOG (rhMOG) and spinal cords were extracted and processed at day 15 (peak of disease). (**A**) Leukocytes were stimulated *ex vivo* with PMA/Ionomycin, and brefeldin A was added in the last 4 hours. Cells were then subjected to surface and intracellular cytokine staining and gated as shown. (**B**) Leukocytes were analysed directly *ex vivo* for the expression of B220, CD19, IgM and IgD. Gating strategy is shown.
(TIF)

Figure S3 **Raw O.D. data for anti-MOG ELISAs.** WT and AID^−/−^ mice were immunized with recombinant human MOG (rhMOG) and titres of anti-MOG Ab were evaluated at day 15 (peak of disease). Raw ODs are shown here and compared with unimmunized mice. Each line represents a separate mouse. In this experiment, 5–6 mice per group were tested and an additional experiment tested 8–12 mice.
(TIF)

Figure S4 **T cells in the CNS of AID^−/−^ do not efficiently produce pro-inflammatory cytokines.** (**A**) WT and AID^−/−^ mice were immunized with recombinant human MOG (rhMOG) and spinal cords were extracted and processed at day 15 (peak of disease). Leukocytes were stimulated *ex vivo* with PMA/Ionomycin, and brefeldin A was added in the last 4 hours. Cells were then subjected to surface and intracellular cytokine staining. Frequency and numbers of cytokine producing cells were tabulated as a percentage of total CD4^+^ cells. This experiment tested n = 8–12 mice per group. (**B**) Clinical scores for this particular experiment are shown. Note that the peak clinical score for the WT mice in this experiment was greater than 10.
(TIF)
