# Supplementary figures and images for: Secondary B Cell Receptor Diversification Is Necessary for T Cell Mediated Neuro-Inflammation during Experimental Autoimmune Encephalomyelitis
Source: PLoS One. 2013 Apr 22;8(4):e61478. doi: 10.1371/journal.pone.0061478 (PMC3632548; doi:10.1371/journal.pone.0061478)

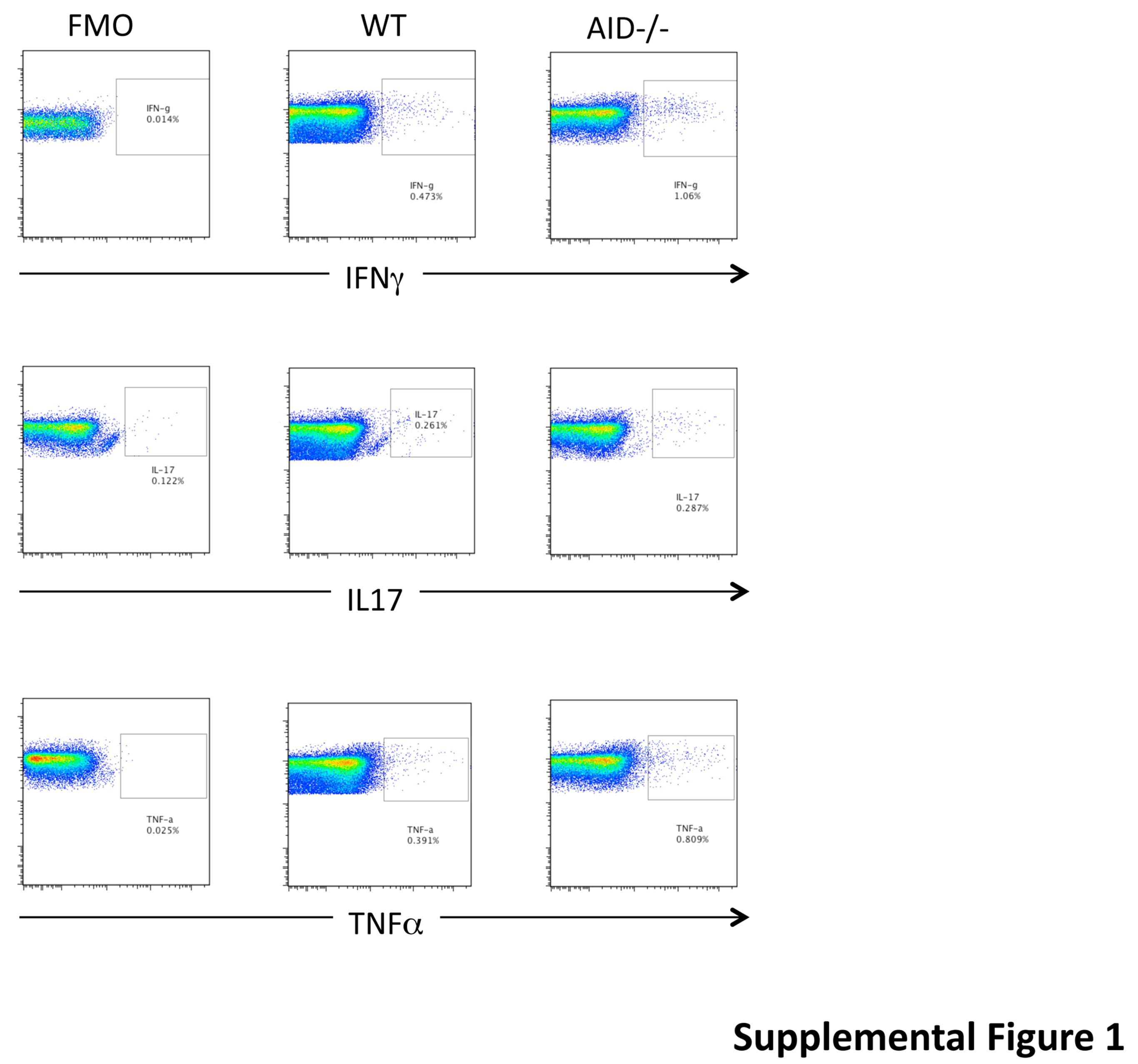

Supplement: Figure S1 — Lymph node CD4+ cells from WT and AID−/− mice both produce cytokines in response to immunization with rhMOG. WT and AID−/− mice were immunized with recombinant human MOG (rhMOG) and draining axillary and brachial lymph nodes were harvested after 7 days post-immunization. 4 million lymph node cells were plated along with 20 µg of rhMOG. Cultures were kept for 48 hours, then were harvested and subjected to intracellular cytokine staining. Shown here are cells pre-gated based on live staining and surface expression of CD4. A representative example is shown and 5 mice per group were tested, with the experiment performed twice with similar results. (TIF) [file pone.0061478.s001.tif]

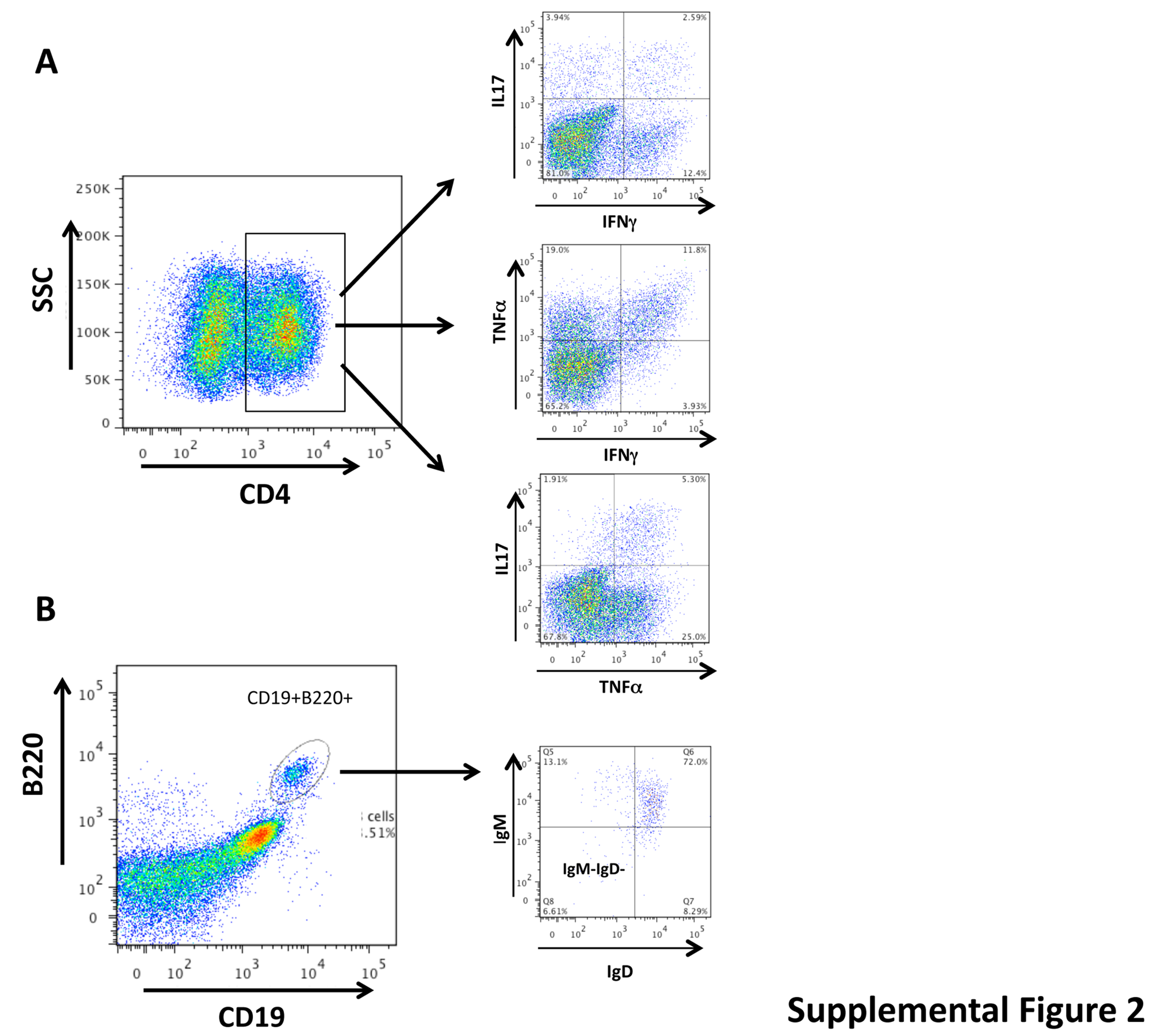

Supplement: Figure S2 — Representative FACS of CNS leukocytes. WT and AID−/− mice were immunized with recombinant human MOG (rhMOG) and spinal cords were extracted and processed at day 15 (peak of disease). (A) Leukocytes were stimulated ex vivo with PMA/Ionomycin, and brefeldin A was added in the last 4 hours. Cells were then subjected to surface and intracellular cytokine staining and gated as shown. (B) Leukocytes were analysed directly ex vivo for the expression of B220, CD19, IgM and IgD. Gating strategy is shown. (TIF) [file pone.0061478.s002.tif]

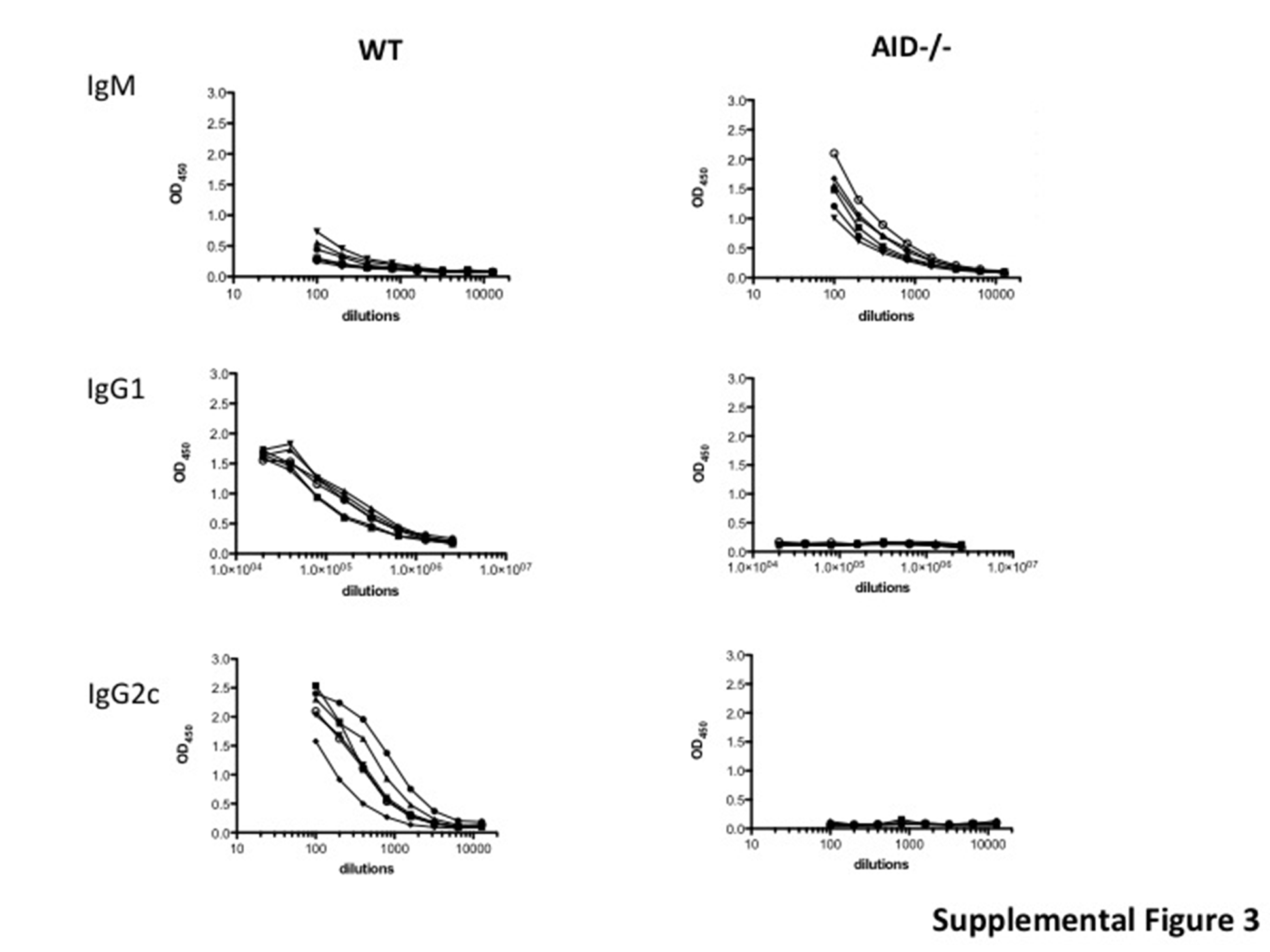

Supplement: Figure S3 — Raw O.D. data for anti-MOG ELISAs. WT and AID−/− mice were immunized with recombinant human MOG (rhMOG) and titres of anti-MOG Ab were evaluated at day 15 (peak of disease). Raw ODs are shown here and compared with unimmunized mice. Each line represents a separate mouse. In this experiment, 5–6 mice per group were tested and an additional experiment tested 8–12 mice. (TIF) [file pone.0061478.s003.tif]

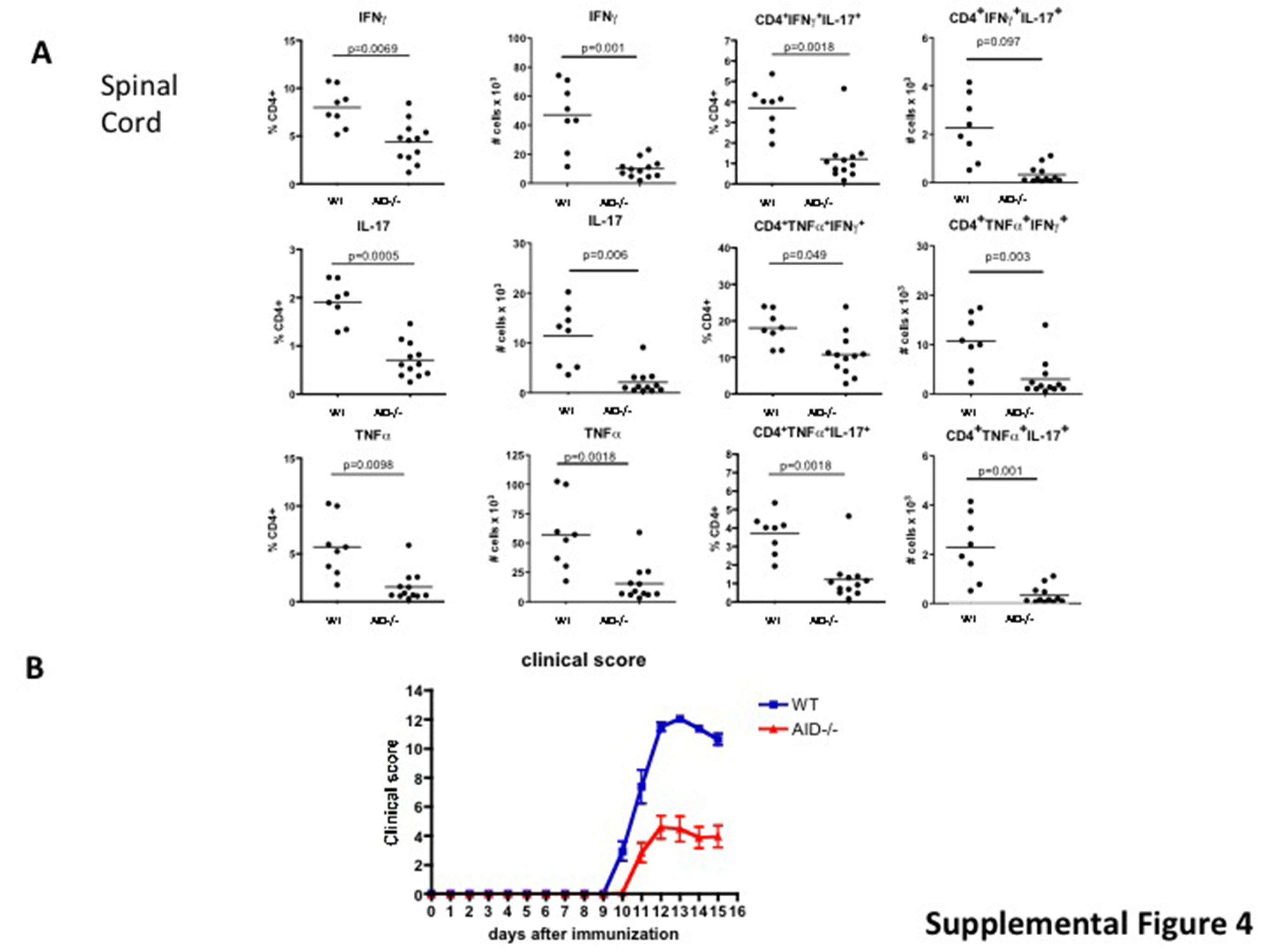

Supplement: Figure S4 — T cells in the CNS of AID−/− do not efficiently produce pro-inflammatory cytokines. (A) WT and AID−/− mice were immunized with recombinant human MOG (rhMOG) and spinal cords were extracted and processed at day 15 (peak of disease). Leukocytes were stimulated ex vivo with PMA/Ionomycin, and brefeldin A was added in the last 4 hours. Cells were then subjected to surface and intracellular cytokine staining. Frequency and numbers of cytokine producing cells were tabulated as a percentage of total CD4+ cells. This experiment tested n = 8–12 mice per group. (B) Clinical scores for this particular experiment are shown. Note that the peak clinical score for the WT mice in this experiment was greater than 10. (TIF) [file pone.0061478.s004.tif]
